# Enzyme phylogenies as markers for the oxidation state of the environment: The case of respiratory arsenate reductase and related enzymes

**DOI:** 10.1186/1471-2148-8-206

**Published:** 2008-07-16

**Authors:** Simon Duval, Anne-Lise Ducluzeau, Wolfgang Nitschke, Barbara Schoepp-Cothenet

**Affiliations:** 1 Laboratoire de Bioénergétique et Ingénierie des Protéines UPR 9036, Institut de Biologie Structurale et Microbiologie, CNRS, F-13402 Marseille Cedex 20, France

## Abstract

**Background:**

Phylogenies of certain bioenergetic enzymes have proved to be useful tools for deducing evolutionary ancestry of bioenergetic pathways and their relationship to geochemical parameters of the environment. Our previous phylogenetic analysis of arsenite oxidase, the molybdopterin enzyme responsible for the biological oxidation of arsenite to arsenate, indicated its probable emergence prior to the Archaea/Bacteria split more than 3 billion years ago, in line with the geochemical fact that arsenite was present in biological habitats on the early Earth. Respiratory arsenate reductase (Arr), another molybdopterin enzyme involved in microbial arsenic metabolism, serves as terminal oxidase, and is thus situated at the opposite end of bioenergetic electron transfer chains as compared to arsenite oxidase. The evolutionary history of the Arr-enzyme has not been studied in detail so far.

**Results:**

We performed a genomic search of genes related to *arrA *coding for the molybdopterin subunit. The multiple alignment of the retrieved sequences served to reconstruct a neighbor-joining phylogeny of Arr and closely related enzymes. Our analysis confirmed the previously proposed proximity of Arr to the cluster of polysulfide/thiosulfate reductases but also unravels a hitherto unrecognized clade even more closely related to Arr. The obtained phylogeny strongly suggests that Arr originated after the Bacteria/Archaea divergence in the domain Bacteria, and was subsequently laterally distributed within this domain. It further more indicates that, as a result of accumulation of arsenate in the environment, an enzyme related to polysulfide reductase and not to arsenite oxidase has evolved into Arr.

**Conclusion:**

These findings are paleogeochemically rationalized by the fact that the accumulation of arsenate over arsenite required the increase in oxidation state of the environment brought about by oxygenic photosynthesis.

## Background

Prokaryotes are able to use a plethora of organic and inorganic substances as electron donating/accepting substrates for their bioenergetic metabolisms. In contrast to the substrate versatility of these bioenergetic chains stands the relatively monotonous architecture of the enzymes involved. These enzymes appear as variations on only a small number of fundamental themes [1] and it seems that many if not most of these enzymes and even complete bioenergetic chains are almost as old as life itself [2-4]. The enormous versatility of bioenergetic metabolism makes particular sense in the first billion years of life on Earth, prior to the rise of molecular oxygen in the biosphere, i.e. before this large reservoir of bioenergetic fuel for aerobic respiration was available.

Arsenics are notorious for their toxicity. This deleterious property nonwithstanding, reduced (arsenite) and oxidized (arsenate) arsenics are recruited by prokaryotes as bioenergetic fuel. Two enzyme systems are presently known to mediate the bioenergetic use of arsenics, i.e. arsenite oxidase and respiratory arsenate reductase. A confusing multitude of acronyms denoting these two enzymes can be found in the literature, whereof Aro (for Arsenite oxidase) and Arr (for Arsenate reductase) appear as the most sensible couple of acronyms to us. These denominations are therefore used throughout this work. The enzyme Aro serves as entry point for reducing equivalents issued from the oxidation of the arsenic oxianion arsenite (As^III^) to arsenate (As^V^) into bioenergetic chains. The enzyme Arr, by contrast, plays the role of a terminal oxidase in prokaryotic energy conversion (for reviews, see [5-8]) and is thus situated at the opposite end of bioenergetic electron transfer chains with respect to its "sister-enzyme" Aro.

Both Aro and Arr are members of a large group of molybdopterin-containing enzymes, commonly referred to as the DMSO superfamily. This superfamily is a textbook example of the above mentioned construction-kit type variation on a theme. The large catalytic molybdopterin-subunit is present in all members of the DMSO superfamily supplemented by a variable number of additional subunits to spawn the multitude of individual subfamilies. In arsenite oxidase, the unique additional subunit is a [2Fe-2S] protein belonging to the family of Rieske-proteins [9].

We have previously studied the phylogeny of both subunits from arsenite oxidase [4-10] and have proposed on the basis of these results that this enzyme has emerged prior to the Archaea/Bacteria split more than 3 billions years ago. Such an early emergence of an enzyme system using arsenite as electron donor for bioenergetic chains makes sense since arsenite is likely to have been present in substantial amounts in the habitats of the early Earth.

Microbes capable of respiring arsenate have been discovered more than a decade ago [11,12]. A molybdopterin enzyme responsible for this metabolic capacity was subsequently described and biochemically analysed in five different arsenate reducing Eubacteria [13-17]. More recently, the corresponding enzyme in *Shewanella *sp. ANA-3 was shown to be encoded by the "*arr*" gene cluster [13] and the presence of *arr*-genes was demonstrated by *arrA *amplification in 13 arsenate-respiring bacteria [18].

The typical *arr *operon contains *arrA *and *arrB *genes coding for hydrophilic proteins with molecular weights of ~96 kDa and ~26 kDa, respectively [13,15]. Based on the lack of a membrane-anchoring subunit in most known cases and the presence of a fully conserved twin arginine translocation (TAT) signal in ArrA sequences, respiratory arsenate reductase has been proposed to be a periplasmic enzyme and to be in most cases soluble (reviewed by Stolz et al. [8]). Whereas the *Chrysiogenes arsenatis *enzyme [15] indeed was found in the soluble fraction, in *Bacillus selenitireducens *the arsenate reductase had to be purified from membrane fractions [16]. In both cases, the purified enzyme only contained the A and B subunits. However, several cases of *arr *operons containing a gene coding for a putative membrane protein have been reported (see Stolz et al. [8]) suggesting a possible role of these proteins for membrane-association of arsenate reductase in selected cases.

The cofactor composition of the enzyme has been predicted from amino acid sequences and was confirmed by metal analyses [15,16]. The amino acid sequence of ArrA features two cofactor binding motifs: a cysteine-rich stretch at the N-terminus predicted to coordinate an iron-sulfur cluster and a molybdopterin dinucleotide-binding domain. The amino-acid sequence of ArrB indicates the presence of four cubane-type iron-sulfur clusters (reviewed in Stolz et al. [8]). With respect to subunit composition, arsenate reductase is thus significantly different from arsenite oxidase which contains a structurally completely unrelated [2Fe-2S]-protein (see above) rather than the tetracubane-type ferredoxin subunit ArrB. In line with this difference, previously published sequence comparisons indicated a quite distant relationship between arsenite oxidase and arsenate reductase with respect to their Molybdopterin-subunits [7,8,18].

Such strong divergence between these enzymes is intriguing considering that the reaction of the catalytic site in the two molybdopterin enzymes is the same, only in reverse. No comprehensive phylogenetic study of the enzyme and its relationship to other closely related subfamilies of the DMSO reductase superfamily has been performed so far. Previously reported phylogenies either considered only a few members of the DMSO superfamily (when dealing with Arr sequences [8]) or completely disregarded *arr *genes (when dealing with a more general phylogeny of the DMSO superfamily [19,20]). To gain further insight into the evolutionary history of respiratory arsenate reductase, we performed an exhaustive genomic search of genes related to *arrA *and reconstructed the overall phylogeny of arsenate reductase, arsenite oxidase as well as further genes closely related to these enzymes.

Our genomic survey firmly established the previously proposed proximity of arsenate reductase to the family of sulfide reductases but also unraveled the existence of a novel clade even more closely related to arsenate reductase, the physiological function of which is still unknown. The obtained tree topologies demonstrate that, unlike arsenite oxidase, the respiratory arsenate reductase is a more recent invention of the bacterial subtree and most probably derives from an enzyme involved in sulfur metabolism. The implications concerning paleogeochemical availability of respiratory substrates as a function of the oxidation state of the environment are discussed.

## Results

### Data mining and sequence analyses

**Figure 1:**
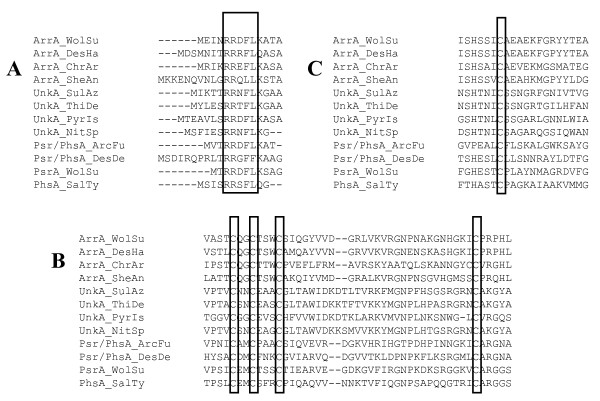
**Conserved motifs encountered in the sequences of the molybdo-subunits ArrA, UnkA or Psr/PhsA**. **a**. To the exeption of one subclass of the Unk sequences (see the text for more details), all three clades feature the TAT (twin-arginine translocation) signal sequence at the N-terminus. **b**. All three types of molybdo-subunits contain the motif for binding an iron-sulfur cluster (C-X_2_-C-X_3_-C-X_27_-C) near the N-terminus. **c**. Amino acid sequence alignment of the molybdenum-binding domain indicates a conserved cysteine residue which is likely to represent the amino acid that coordinates to the molybdenum.

When we initiated this study, only eleven *arrA *sequences from 26 arsenate respiring species were available. In the four species *Bacillus arseniciselenatis, Bacillus selenitireducens, Chrysiogenes arsenatis, Sulfurospirillum barnesii *and *Desulfosporosinus *sp. strains Y5 and DCB-2, only the *arrA *gene has been sequenced (by PCR see [18,21]) and the subunit composition of the enzyme is unknown. Only six *Arr *operons were therefore available in previous analyses [8]. Starting from this set of sequences, we searched for further homologous genes in whole genome databases using *arrA *from *Shewanella *ANA-3 as query sequence. This search yielded a multitude of putative molybdopterin-proteins. Only genes located near putative *arrB *sequences (homologous to Shewanella *arrB*) and possessing the three conserved motifs (shown in Figure 1), *i.e*. the TAT signal sequence, the molybdopterin binding domain, and the [4Fe-4S] binding domain, were retained in a first selection step. Intriguingly, many so-defined gene clusters contained a third gene adjacent to the *arrAB *diade. This gene was already noticed previously and, due to its relatedness to the *psrC *gene of polysulfide reductase (see below), denoted as "*arrC*".

It is noteworthy that several of the species described to respire arsenate lack the *arrA *gene [18] and their metabolic capacity to respire arsenate is therefore probably conferred by a different, hitherto unknown, enzyme.

As has already been noticed in the past [15,16,18,21], BLAST scores indicate strong similarities between the catalytic A-subunits of Arr and of the enzyme polysulfide reductase (Psr) and we therefore included Psr gene clusters in our data searches. However, since Psr is not the focus of this work, only selected representatives of Psr were considered for tree reconstructions covering as much as possible the phylogenetic tree of species. BLAST searches with PsrA from *Wolinella succinogenes *[22] as query sequence recognized all *arrA *genes already retrieved *via *ArrA from *Shewanella *ANA-3 but also yielded further genes as discussed below. BLAST scores are therefore insufficient to reliably distinguish between *arrA *and *psrA*. However, as will be shown below, both the phylogeny of the catalytic subunit and the distinctive organisation of gene clusters allow an unambiguous identification of the retrieved genes.

A further enzyme involved in bioenergetic sulfur metabolism and containing the three canonical subunits A, B and C has been described in the literature, *i*.*e*. the tetrathionate reductase (Ttr) from *Salmonella typhimurium *[23]. High sequence similarities between the A-subunits from Ttr and Psr have been noticed previously [23-26]. We therefore decided to include Ttr sequences in our genome mining approach. Using *ttrA *from *Salmonella typhimurium *as query sequence, we searched for homologous genes in whole genome databases. This search yielded a multitude of putative TtrA proteins from which representative cases were included in the phylogenetic reconstruction.

In addition to sequences which are closely related to known arsenate reductases, we added the so far known cases of arsenite oxidases [4,8], replenished with new sequences retrieved during our present genomic survey. We would like to emphasize that the partial AroA sequences reported by Inskeep *et al*. [27] were not included in our tree reconstruction. These sequences represent only a small fraction of the full-length protein and including these partial ORFs substantially lowers the reliability of the obtained trees. Selected representatives of the "standard" molybdopterin enzymes formate dehydrogenase [28,29], DMSO reductase [30] and nitrate reductase [31,32] have furthermore been included in the set of sequences used for tree reconstruction (all protein accession numbers, related to its related enzyme and strain are listed in the Additional file 1). For the DMSO reductase case, only representatives containing the iron sulfur binding motif in the N-terminal domain of the molybdopterin subunit [30] were considered.

### Phylogeny of the catalytic subunit and gene cluster organisation

**Figure 2:**
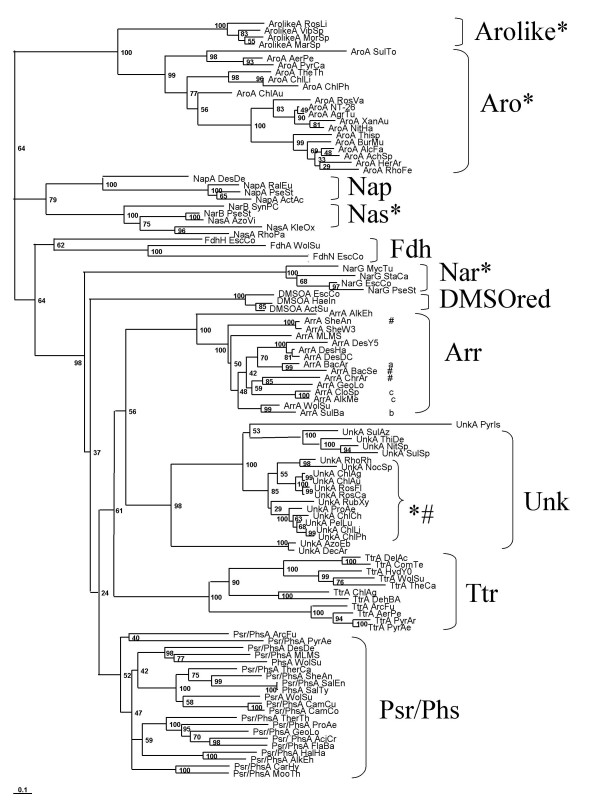
**NJ-phylogram of the molybdenum subunits of the DMSO reductase family**. Almost all the represented enzymes harbor the TAT signal of export to the periplasm. Enzymes lacking this signal are marked by "*". This is the case of entire families (Nas, Nar, Aro or FdhH) or subfamilies (Unk). Arsenite oxidase (Aro) forms a separate and distant clade and is therefore used as outgroup. Arsenate reductase (Arr), the new uncharacterized molybdoenzyme (Unk, see the text), tetrathionate reductase (Ttr) and polysulfide reductase/thiosulfate reductase (Psr/Phs) form distinct but related clades. Whereas representatives of the Psr/Phs clade all contain the anchor subunit, some representatives of the Unk and Arr clades (marked with #) were lacking this subunit. ^a^Only the sequence of the A subunit of *Bacillus arsenicoselenatis *is known. ^b^The Arr of *Sulfurospirillum barnesii *has been demonstrated biochemically to be composed of three subunits but only the A subunit has been sequenced. ^c^The Arr of *Alkaliphilus oremlandii *(formely *Clostridium *sp.) OhILAs and *Alkaliphilus metalliredigenes *have been published as composed of three subunits. We failed, however, to detect a typical ArrC subunit in the Arr operon. On the other hand our study revealed Arr operons in *Geobacter lovleyi SZ*, *Shewanella *sp. W3-18-1 and *Alkalilimicola ehrlichei *MLHE-1, the two first without and the last with the third subunit C, respectively.

**Figure 3:**
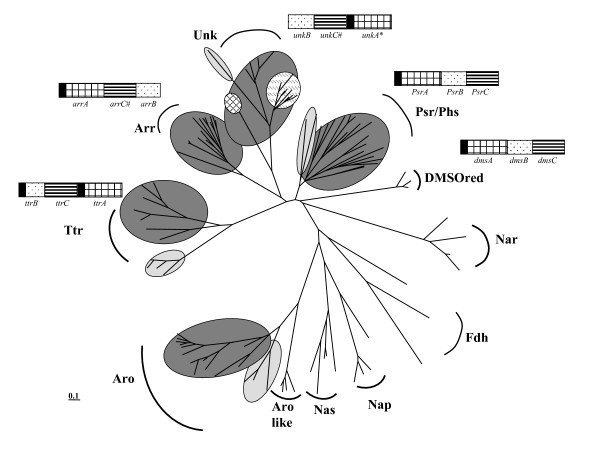
**Schematic phylogenetic (unrooted neighbor joining) tree of the molybdenum subunits of the DMSO reductase superfamily**. Arsenite oxidase (Aro) forms a clade distant from arsenate reductases (Arr) with the dichotomy Bacteria (in dark grey)/Archaea (in light grey) indicating that the origin of this enzyme pre-dates the divergence of domains. The same dichotomy is also observed within the Psr/Phs and the Ttr clades. A corresponding dichotomy is less obvious for the Unk clade and clearly absent in the Arr family. The operons of all four families: Psr/Phs, Ttr, Unk and Arr, have three genes in common denoted A, B and C, coding for the molybdo-(with the TAT signal marked in black), iron-sulfur- and anchor-subunits, respectively. These three genes are arranged in the Psr/Phs operon in the same order as in the *Escherichia coli dms *operon, i.e. ABC. In the case of the *arr *operon the order is CAB, C occasionnally missing (marked by #). In the case of the *ttr *and *unk *(when C is present) operon, the order is BCA. In this family a subgroup (highlighted by the hatched circle), lacks the C subunit and the TAT signal on the A subunit (marked by*) and contains abnormally large subunit B and therefore possibly represents a separate type of enzyme.

The phylogenetic tree reconstructed from sequences of the catalytic molybdopterin subunit (see Additional file 2) via the Neighbor-Joining (NJ)-method is shown in Fig. 2. In addition to the well-known clusters formed by DMSO and nitrate reductases as well as formate dehydrogenases, the tree features 6 well-defined clades (Figs. 2 and 3). The reliability of these clades is supported by their coincidence with an evolutionary marker, *i*.*e*. the gene organisation of A, B and C genes within the respective gene clusters. As indicated in Fig. 3, the order of these three genes as well as the location of the TAT motif varies between clades but is conserved within each individual clade.

The Arr clade is characterized by either *arrCAB *or *arrAB *gene clusters, *i*.*e*. positioning the gene encoding the membrane anchor protein upstream of the arrAB diade or completely lacking this gene (branches indicated by # on Fig. 2). No archaeal Arr representatives could be detected and the retrieved sequences in fact arise exclusively from δ-, ε-, γ-proteobacteria, Firmicutes and Chlostridia. The topology of the Arr subtree differs significantly from that of species trees. Despite specific efforts, we only found three new unambiguous *arrA *sequences *i.e*. those from *Alkalilimnicola ehrlichii*, *Geobacter lovleyi SZ*, and *Shewanella *sp. W3-18-1. Gene clusters coding for respiratory arsenate reductases therefore appear to be rare as compared to those of their sister enzymes (see below). A tentative rationalisation for this observation will be given in the Discussion Section.

As mentioned above, the Psr enzyme is often cited as closest relative of respiratory arsenate reductases. Polysulfide reductases (Psr) perform the respiratory conversion of elemental sulfur to H_2_S. Since S° is rather insoluble in aqueous solutions, an alternative form of sulfur, polysulfide, is generally considered to be the actual substrate for sulfur respiration. Polysulfide reductase from *Wolinella succinogenes *was the first enzyme from this class to be sequenced [21] and homologous genes have been identified more recently in the Archaea *Pyrobaculum aerophilum *[33] and *Archaeoglobus fulgidus *[34]. In addition to the molybdopterin catalytic subunit PsrA and the tetracubane ferredoxin PsrB, polysulfide reductase contains a third subunit denoted PsrC. PsrC is a membrane-integral protein and is considered to serve as membrane anchor for the extrinsic subunits PsrA and PsrB. The PsrC protein seems to harbor the site where quinol is oxidized and thus to furnish reducing equivalents ultimately consumed in the reduction of polysulfide [35]. PsrC therefore is an indispensable part of the whole enzyme and is consequently present in all Psr gene clusters.

In the Psr clade, gene clusters are arranged in the order ABC, *i*.*e*. the gene coding for the catalytic molybdopterin subunit is followed by that encoding the tetracubane iron sulfur protein whereas the gene of the membrane-integral protein comes last (Fig. 3). In contrast to the situation in the Arr cluster, the *psrC *gene is present in all branches of the Psr clade. This operon organisation is by the way similar to that of DMSO reductase (*dmsABC*). The congruence of phylogenetic grouping and gene cluster structure (Fig. 3) strongly corroborates the attribution of function suggested by the observed clade. The genes yielding several of the branches in the Psr clade are not annotated in accordance with the clade, that is the Psr clade contains members annotated as *arrA *or even featuring more exotic attributions suggesting the necessity of reannotating the aberrantly denoted genes.

It is noteworthy that one branch within the clade denoted "Psr" in fact represents an enzyme which has been biochemically characterized in *Salmonella typhimurium *to be a thiosulfate reductase (Phs) rather than a polysulfide reductase [36]. The gene cluster encoding this enzyme in *Salmonella typhimurium *features an "ABC-order", just as the *bona fide *Psr cases do. Both Psr and Phs use electrons from the quinone pool to reduce S° and S_2_O_3_^2-^, respectively, to H_2_S. These enzymatically very similar reactions thus seem to be catalysed by evolutionarily closely related enzymes. The ensuing high sequence similarities between the catalytic subunits of polysulfide and thiosulfate reductases render a straightforward classification of genes retrieved in our genome mining into one or the other group of enzymes difficult. A possible clue for this purpose may reside in the length of the C-subunit. Whereas PsrC from the biochemically characterized polysulfide reductase from *Wolinella succinogenes *has 8 predicted transmembrane (TM) helices, only 5 TM stretches are observed in PhsC from the thiosulfate reductase of *Salmonella typhimurium*. According to this criterion, the branches labeled *DesDe*, *SalEn*, *HalHa *and *AlkEh *would be thiosulfate rather than polysulfide reductases. However, only a detailed functional characterization of all members of this subclade will definitively settle this question and we therefore opted to denote the corresponding branches as *psr*/*phs*.

The Psr/Phs clade is characterized by a profound cleavage into archaeal and bacterial branches suggesting an origin of this enzyme system prior to the divergence of domains. In contrast to the notion frequently put forward that Psr is most closely related to Arr, two additional clades are observed in Figs. 2 and 3 in between the arsenate and the polysulfide/thiosulfate reductase clusters. One of these clades contains the biochemically characterized tetrathionate reductase (Ttr) from *Salmonella typhimurium *and we therefore call this subtree the Ttr clade. All enzymes constituting this clade are encoded by gene clusters featuring the order BCA and an additional TAT signal on the B subunit [26], differing from all other clades discussed so far. Similar to the Psr clade, however, the Ttr subtree shows a deep bifurcation into archaeal and bacterial branches indicating an origin of this enzyme within the common ancestor of the two prokaryotic domains.

A subset of sequences retrieved in this genome survey forms a clade which contains neither *bona fide *Arr, nor Psr/Phs, nor Ttr enzymes. Once again, this clade features a conserved structure of its encoding gene cluster which differentiates it from the *arr *and *psr/phs *classes. The AB diade is inverted in this clade and in some cases is separated by the membrane anchor protein yielding a BCA order of genes (Fig. 3), i.e. reminiscent of that seen in the Ttr clade. In this group of sequences, all cases lacking the membrane-anchor protein cluster together (marked by # in Fig. 2), contain a larger tetracubane subunit (60 kDa instead of 26 kDa) and lack the TAT signal sequence (marked by * in Fig. 2) in the molybdopterin subunit. These cases therefore form a distinct subgroup. Moreover, two long proteobacterial branches (*Azoarcus sp*. EbN1 and *Dechloromonas aromatica *RCB) appear as outgroup to the remaining sequences in this group. A detailed inspection of their A and B subunits' sequences clearly establishes their divergence from the main group. This clade therefore likely represents three distinct types of enzymes. For none of the branches in this clade, the encoded enzymes have been studied so far. The metabolic function/functions of this class of enzymes is/are thus so far unknown and we correspondingly chose to label the respective genes as *unkA*, *unkB *and *unkC*.

With respect to the previously studied set of arsenite oxidases (Aro) [4,8], our present genome survey detected several new genes strongly similar to those of Aro. Four of these new sequences in fact do not cluster within the Aro-clade but branch off close to this family. Although in all four cases, the gene coding for a Rieske protein is present upstream of the molybdenum-subunit, just as in genuine Aro-operons, their gene clusters feature several conserved ORFs not present in Aro operons. Furthermore, this set of four sequences differs significantly from those of the Aro cluster by several distinctive stretches of residues. The fact that none of the parent species has been described to perform the oxidation of arsenite indicates that this clade represents a new subclass of molybdopterin-enzymes with hitherto unknown function and we correspondingly denote the catalytic subunits of this clade as as "ArolikeA".

With respect to the genuine Aro subtree, the phylogeny shown in Figures 2 and 3 corroborates the conclusions arrived at in our previous work suggesting this enzyme to have already been present in the Last Universal Common Ancestor (see [4]). An early origin of arsenite oxidase is also predicted by the position of its Rieske subunit on a tree encompassing arsenite oxidases and Rieske/cyt*b *complexes [10]. The unrooted tree shown in Fig. 3 thus strongly suggests the clade containing Aro and the closely related enzyme of unknown function (Arolike) as outgroup to the other molybdopterin enzymes. This scenario contradicts that published by McEwan et al. [19] considering arsenite oxidases as having evolved from a Nas/Nap/Fdh-type ancestor.

As discussed above, the phylogenetic trees shown in Fig. 2 and 3 are based on the sequences of the catalytic molybdopterin subunit, *i.e*. the A-subunit. Since Arr, Psr/Phs, Ttr and Unk all contain the tetracubane iron-sulfur (B-) as well as the membrane-integral C-subunit, it may appear tempting to corroborate the A-phylogeny by those obtained from these additional subunits. However, as we have already stated in a previous article [1] and as is obvious from the results obtained by Rothery *et al *[20], these two subunits are ill-suited for phylogenetic studies. The B-subunit is a small protein of about 22 kDa featuring a high number of strictly conserved residues necessary for ligating the four iron-sulfur clusters (cysteines) and maintaining the required loop structure (prolines). The number of phylogenetically informative sites is therefore small and insufficient to resolve the long evolutionary distances relevant to our work. The C-subunit, also being much smaller than the A-subunit, is complicated by its complete absence of fully conserved residues and by its variable sequence length. Reliable multiple alignments of C-subunit sequences are difficult to attain as long as no 3D-structures are available.

### The membrane-integral C-subunit

The "C"-genes retrieved for members of the Arr, Psr/Phs and Unk-families all code for hydrophobic proteins of about 30 kDa containing at least 5 predicted TM helices. Members from all three families are obtained with significant expectation values in BLAST searches using any of the individual ArrC, Psr/PhsC and UnkC sequences as queries and the *dmsC *gene of DMSO reductases is also recognized. Multiple sequence alignments show that the members of all four families are indeed related with significant sequence similarities in the C-terminal part of the protein (not shown), but in contrast to what is observed for the molybdopterin subunit, no fully conserved residue positions are detected if all four families are compared.

The membrane-integral C-subunits of Arr, Psr/Phs, Unk, Ttr and DMSO reductase thus probably represent a common module employed by these molybdopterin enzymes for the purpose of membrane attachment and, most probably, mediating electron exchange with the quinone pool. The absence of conserved residues such as histidines argues against the presence of heme cofactors in these C-subunits, contrary to what is claimed in some annotations. Correspondingly, in none of the studied systems, experimental evidence for heme prosthetic groups in these enzyme complexes has been reported. The C-subunits of these five families thus differ from other groups of the molybdopterin superfamily, which can contain small helix-bundle diheme *b*-type cytochromes (formate dehydrogenase and Nar nitrate reductase, [29,37-39]) or membrane-tethered globular tetraheme *c*-type cytochromes (called the NapC/Nir-family, see for example [40]) to connect the intra-enzyme electron transfer to the quinone pool.

## Discussion

### Arsenite oxidase and respiratory arsenate reductase are phylogenetically only distantly related

Three enzyme systems are so far known to mediate redox conversions of arsenics. One of them, *i*.*e*. the so-called arsC/acr2-system, serve a purely detoxifying function, is cytoplasmic and consumes ATP during the detoxifying reduction (and extrusion from the cytoplasm) of arsenic [for recent reviews see [5,41]]. The corresponding enzymes do not contain metal-cofactors and are evolutionarily unrelated to the two systems dealt with in this work, *i*.*e*. the bioenergetic enzymes arsenite oxidase and respiratory arsenate reductase.

Arsenite oxidase is generally considered to catalyse the two-electron oxidation reaction of As^III ^to As^V ^and to pass the reducing equivalents on to soluble periplasmic electron shuttle proteins, such as cytochromes or copper proteins [42-44]. The more recently discovered enzyme respiratory arsenate reductase is thought to perform this reaction in reverse. Although both enzymes indeed use molybdopterin proteins from the DMSO reductase superfamily to catalyze this reaction, their subunit composition differs substantially with Arr containing a tetracubane Fe-S protein rather than the Rieske subunit of Aro as second subunit in the enzyme complex. Previous phylogenetic analyses based on a small sample of sequences also indicated a rather distant relationship between these two enzymes [8]. In order to better understand these puzzling findings, we screened available genomes for gene clusters related to arsenate reductase. The results detailed above fully confirm the absence of a direct evolutionary link between Aro and Arr.

Respiratory arsenate reductase instead forms a compact phylogenetic clade which is most closely related to an enzyme of so-far unknown function the representatives of which also cluster together in a tight clade. Both these clades are phylogenetically close to the groups of polysulfide/thiosulfate (Psr/Phs) and tetrathionate reductases (Ttr). Sequence homologies between Psr/Phs and Ttr have already been emphasized [23-25].

### Deducing evolutionary histories from the comparison of enzyme and species phylogenies

As already discussed in previous articles [4,10] and corroborated by the extended data set of the present work, phylogenies of both subunits of arsenite oxidase closely match those of the parent species. Both archaeal and bacterial representatives of the enzyme have been detected and the two domains are well-separated on phylogenetic trees of Aro's molybdenum and Rieske subunits. This enzyme complex thus shows distinctive characteristics suggesting its evolutionary origin to pre-date the Archaea/Bacteria cleavage some 3 billion years ago.

The situation is radically different for the phylogeny obtained for Arr (Fig. 2). On the Arr subtree, (δ-, ε- and γ-) proteobacterial sequences are intermingled with those from Firmicutes and Chlostridia. Even the various proteobacterial lineages do not cluster together and δ-, ε- and γ-proteobacterial representatives seem almost erratically scattered over this subtree. Despite specific effort to this end, we were unable to find any archaeal members of this family. This result is in accordance with the result of Malasarn *et al*. [18] who failed to amplify *arrA *genes in archaeal As^V ^respiring species. The phylogeny of Arr thus provides a textbook example of an enzyme distributed over a range of species largely via horizontal gene transfer. The presently available sample of sequences would suggest an origin in a γ-proteobacterium followed by dispersal over the other observed phyla. However, with increasing numbers of genomes containing *arr *gene clusters, this specific scenario may well require modifications.

The detailed affiliation of the Arr subtree to those of the Psr/Phs, Ttr and Unk-enzymes provides hints for the evolutionary origin of arsenate reductase. The overall tree topology suggests that all three, Arr, Ttr and Unk, are derived from an enzyme closely related to the ancestor of Psr/Phs. Our genome search suggests that Ttr, originally considered to be specific to Enterobacteriaceae [23], is widely distributed among Archaea and Bacteria. Our results futhermore suggest an early origin of this enzyme, prior to the Archaea/Bacteria split. For the case of Arr, the root of the enzyme appears to lie in the bacterial domain according to the presently available ensemble of sequences. The case is less clear-cut for the case of the Unk enzyme, since in the absence of any biochemical/molecular biological evidence we propose to consider this group as three distinct clades. Biochemical characterization of selected enzymes is thus required before the clade previously called Unk can be interpreted properly.

We would like to note that in many previous articles dealing with phylogenies of members of the DMSO superfamily [8,20], the general aspect of trees differs substantially from that of our tree by the predominance of deep branching nodes and very long branches. Although no respective information is provided in the Materials and Methods Sections of these articles, we strongly suspect that the corresponding trees were reconstructed neglecting the possibility of multiple substitutions. Regarding the enormous evolutionary distances between the respective enzymes, we consider this approach as erroneous and would insist on the fact that multiple substitutions need to be allowed for.

Sulfur respiration and the related enzymes are widespread among Bacteria and Archaea [24]. In aqueous solution, sulfur occurs in a variety of compounds including polysulfides and polythionates. Diverse organisms are able to use these sulfur species as respiratory electron acceptors by reductively cleaving the sulfur-sulfur bonds. In many cases, the enzymes catalysing these reactions contain a bis(molybdopterin guanine dinucleotide)molybdenum (MGD) cofactor at the catalytic site. The best studied examples of such enzymes are the tetrathionate (Ttr) [35,46], thiosulfate (Phs) [35] and polysulfide reductases (Psr) [24]. It has been demonstrated that, in *Salmonella enterica*, which contains both Ttr and Phs, Phs was unable to restore a tetrathionate reduction in a *ttr*-mutant [25] but reduced polysulfide. This result is in accordance with the obtained phylogenetic tree suggesting that Psr and Phs are phylogenetically much more closely related to each other than to Ttr. Corresponding cross-reactivities of Arr and Phs/Psr towards thiosulfate, polysulfide and arsenate have not been studied so far. However, it is interesting to note that Phs is capable of reducing chlorate to chlorite [45]. This reduction involves an oxygen atom transfer, just as the reduction of arsenate to arsenite, rather than the sulfur-sulfur bond cleavage of thiosulfate reduction. It therefore is tempting to consider that Phs/Psr may by themselves be able to perform arsenate reduction. The observed sequence homologies indeed suggest that arsenate reductase evolved from a Phs/Psr ancestor. Rather than reversing the reaction mechanism of arsenite oxidase, evolution modified an enzyme of the sulfur respiration pathway, the ancestor of the Psr/Phs, to create the arsenate reductase.

### Paleogeochemical and bioenergetic constraints rationalize the differing evolutionary histories of arsenate reductase and related enzymes

Reduced arsenics such as arsenite are present in significant quantities in modern hydrothermal environments and are likely to have been so throughout Earth's history back to the Archaean and Hadean era. It thus isn't astonishing that life should have used this abundantly available source of reducing equivalents via arsenite oxidase as electron donor to bioenergetic electron transfer chains already in its early days.

In extant environments, the conversion of As^III ^to As^V ^requires the presence of molecular oxygen. O_2_, however, is considered to have been virtually absent in the archaean world, *i*.*e*. prior to its accumulation via oxygenic photosynthesis. Paleogeochemical evidence shows that molecular oxygen becomes detectable in the biosphere of our planet by 2.4 Ga ago [46]. Almost certainly, oxygenic photosynthesis has produced O_2 _long before that date [47]. The time span needed to exhaust the reservoir of reductant (mostly Fe^2+^) in the biosphere before net O_2 _started to increase is a matter of dispute. It seems likely, though, that prior to the Bacteria/Archaea cleavage, no O_2 _was produced photosynthetically [48]. During this period of Earth's history, the ambient potential of the environment was thus much lower than today and biological oxidation of arsenite to arsenate was probably the only mechanism producing oxidized arsenics. Moreover, the above mentioned reductant reservoir will have rereduced the arsenate formed by such oxidation mechanisms. Substantial accumulation of arsenate had to await the increase of the oxidation state of the biosphere brought about by oxygenic photosynthesis. Only at that moment, arsenate turned into a potential substrate for bioenergetic chains by serving as terminal electron acceptor. Based on the phylogenies we observe on the respective enzymes, it seems likely that at this time, an enzyme related to polysulfide reductase has evolved into an arsenate reductase. The substantially lower redox potential of thiosulfate (${\text{E}}_{0}{'}_{\left({\text{S}}_{2}{\text{O}}_{\text{3}}^{\text{2-}}/{\text{HS}}^{-}\right)}$ = -400 mV), and sulfur (${\text{E}}_{0}{'}_{\left({\text{S}}^{\text{0}}/{\text{HS}}^{-}\right)}$ = -275 mV), as compared to that of the As^V^/As^III ^couple (E_m _= +60 mV) suggests that oxidized sulfur compounds were available as electron acceptors already at much lower oxidation states of the environment, *i*.*e*. much earlier than the arsenics. This fact rationalizes the evolutionary ancestry of sulfur-reducing mechanisms, as suggested by the Psr/Phs phylogeny shown in Fig. 2, over that involving the reduction of arsenate.

In this context, the case of the tetrathionate reductase is intringuing. The phylogeny of this enzyme as shown in Figure 2 clearly suggests a pre-divergence origin. Chemically, the most straightforward way to produce tetrathionate is via thiosulfate. The midpoint potential of this redox couple is relatively high (${\text{E}}_{0}{'}_{\left({\text{S}}_{4}{\text{O}}_{\text{6}}^{\text{2-}}/{\text{S}}_{2}{\text{O}}_{\text{3}}^{\text{2-}}\right)}$ = +24 mV) and formation of tetrathionate from thiosulfate would thus probably require a relatively high oxidation state of the environment as detailed above for the case of arsenic. However, in contrast to the simple redox couple As^III^/As^V ^of arsenic oxyanions, the chemistry of sulfur is complicated and multiple reaction pathways to arrive at a given compound are accessible. Disproportionation reactions, for example, may account for the production of relatively oxidized sulfur species [49] even at low redox potentials. A more detailed analysis of the enzyme tetrathionate reductase has therefore been initiated in our group.

Considering basic bioenergetic features of respiratory arsenate reduction, the energetic benefit of this mechanism is expected to be much lower than that or the other systems discussed in this work. The oxidized oxyanion of arsenic, i.e. arsenate, has a pK value slightly below 7, whereas the corresponding pK is shifted by two pH-units to more alkaline values in the reduced arsenite. At neutral pH and below, the redox reaction of arsenic thus corresponds to

H_2_AsO_4_^- ^+ 3 H^+ ^+ 2 e^- ^<---> H_3_AsO_3 _+ H_2_O (below pK of arsenate)

HAsO_4_^2- ^+ 4 H^+ ^+ 2 e^- ^<---> H_3_AsO_3 _+ H_2_O (above pK)

Biochemically characterized respiratory arsenate reductases have been shown to be located on the periplasmic side of the membrane [15,16]. This location can most probably be generalized to all members of the family as judged by the presence of the TAT signal in all retrieved ArrA sequences. Such a reduction of arsenate and concommitant consumption of protons in the periplasm is strongly counterproductive for a respiratory enzyme in that it contributes to collapsing rather than generating the proton-motive-force required for ATP synthesis.

The situation is less dramatic if not only the enzyme but also its embeddedness in the bioenergetic chain is taken into account. As discussed above, the presence of the membrane anchor protein ArrC and its strong relatedness to the PsrC subunit of polysulfide reductase, suggest that, in the species where ArrAB is complemented by ArrC, the electrons serving to reduce arsenate are derived from oxidation of a quinol binding at the ArrC subunit. Assuming that the quinol binding site on ArrC was located such that the protons arising from quinol oxidation are ejected into the periplasm, the combined reaction would read as follows:

H_2_AsO_4_^- ^+ H^+ ^+ QH_2 _<---> H_3_AsO_3 _+ H_2_O (below pK of arsenate)

For the species where ArrC is absent, the enzyme appears to be coupled to the quinone pool by other proteins. In *Shewanella *for example, where only ArrA and ArrB are present, a membrane-tethered periplasmic *c*-type cytochrome, CymA, has been shown to be involved in the respiration of arsenate [50]. CymA is also required for respiration of fumarate, nitrate, Fe(III), DMSO and nitrite [51] and thus apparently acts as common branching point between the quinone pool and multiple terminal oxidases. CymA belongs to the NapC/NirT family which has been shown in other enzymes to mediate electron transfer between the liposoluble quinone pool and molybdopterin enzymes. Several different solutions may therefore be employed by different species to connect the two-subunit Arr enzymes to the quinone pool.

With increasing oxygen content of the environment, bioenergetic mechanisms, optimized to the low redox poise of an anoxic Earth, had to adapt to these environmental changes. This task has been achieved in several phyla of the prokaryotes (both Bacteria and Archaea) by increasing the redox potentials of all cofactors involved in the respective energy conserving chains. Such a shift in redox poise of whole chains necessarily implies the replacement of the ancestral low potential (E_m _= -70 mV) menaquinones by higher potential (E_m _= +100 mV) quinones such as ubi-, plasto- or caldariellaquinone [52,53]. As discussed above, the reducing equivalents for the reduction of arsenate appear to be furnished directly by the quinone pool. However, considering the above mentioned midpoint potentials of quinone species and of the arsenate/arsenite couple, a substantial driving force for electron transfer from the quinone pool to arsenate is only expected if the pool is constituted by low potential menaquinones. In line with this reasoning, almost all species containing Arr gene clusters indeed use menaquinone for their liposoluble electron transfer. Even more intriguingly, one species which contains the Arr gene cluster but uses the higher potential ubiquinone, *i*.*e*. *Alkalilimnicola ehrlichii *has been shown to be unable to respire arsenate [54]. Energetic considerations therefore predict that only a very limited set of organisms can use arsenate for bioenergetic ends. These energetic constraints may well rationalize the small number of Arr gene clusters that we were able to detect in published genomes as compared to those of closely related enzyme systems.

## Conclusion

These findings are paleogeochemically rationalized by the fact that the accumulation of arsenate over arsenite required the increase in oxidation state of the environment brought about by oxygenic photosynthesis.

## Methods

Open reading frames (ORFs) coding for subunits of respiratory arsenate reductase, polysulfate reductase and novel molybdopterin enzymes were retrieved from the National Center for Biotechnology Information (55) using the ArrA/B sequences [GenBank:AAQ01672.1 and AAQ01673.1] from *Shewanella *ANA-3 as query templates in BLAST searches. Psr sequences were retrieved by starting from PsrA/B/C sequences [GenBank: NP_906381.1, NP_906382.1 and NP_906383.1] from *Wolinella succinogenes*. Tetrathionate reductase sequences were retrieved by starting from TtrB/C/A [GenBank: NP_460348.1, NP_460350.1 and NP_460349.1] from *Salmonella typhimurium*.

All structures were obtained from the pdb database (56).

Structural alignments were obtained using the root-mean-square fit option of the Swiss-Pdb Viewer (version 3.7;57).

Secondary structure prediction was performed by means of the pSAAM package (58)

ClustalX [59] was used to obtain multiple sequence alignments of MPT/FeS subunits and anchor subunits, followed by manual adjustment guided by structural information when necessary.

Phylogenetic trees were reconstructed from these alignments using the NJ algorithm implemented in ClustalX or using the parsimony method (PHYLYP package).

## Abbreviations

Aro: arsenite oxidase; Arr: arsenate reductase; NJ: Neighbor-Joining; Psr: polysulfide reductase; Phs: thiosulfate reductase; Ttr: tetrathionate reductase.

## Authors' contributions

SD participated in sequences mining and in multiple alignments. ALD carried out the analysis of the *Sulfurihydrogenibium azorense *genome which is not yet accessible for BLAST searches. WN participated in the design of the study helped to analyse the sequences and to draft the manuscript. BSC initiated and developed this study, participated in sequence mining, in sequence alignments, and in writing the manuscript. All authors read and approved the final manuscript.

## Supplementary Material

Additional file 1 Table 1. Description of Mo-pterin subunits used in phylogenetic analyses. Table 1 (Excel spreadsheet: "Table1.xls") presents GenBank accession numbers of Mo-pterin subunits used for the phylogenetic trees of Figures 2 and 3. Each protein (designed with its own acronym) is related to its related enzyme and strain (name and taxonomy). Click here for file

Additional file 2 **Mo_align**. Multiple alignment of the Molybdopterin-containing catalytic subunits considered in this work. Click here for file
